# Neglect of the Study of Insanity by Physicians

**Published:** 1846-06

**Authors:** A. Brigham

**Affiliations:** Superintendant of the New York State Lunatic Asylum


2. *Neglect of the study of Insanity by Physicians.*—Many physicians in general practice are, we think, too apt to consider insanity a disease with which they have but little or nothing to do. Hence they often neglect to qualify themselves, by study and careful observation, to treat it properly. Their attention is not directed to it, the same as to other diseases, by lectures in the medical schools, and they seldom purchase and read the best treatises on the subject.

It is very true, that in most cases, when the disease becomes established, it cannot be well treated at home. Patients often become prejudiced against their nearest friends, and will not hearken to their advice nor take any medicine. For these reasons and to remove them from the exciting causes of their disease, as well as to have them treated properly in all respects, it is usually necessary to place them in an asylum. Still there are instances, though they are not common, when none of these objections nor any other exist to the patient remaining at home, and being treated by his ordinary physician. It is therefore very necessary that physicians in general practice, should qualify themselves to prescribe in cases of insanity, as well as in other diseases.

But admitting that no such cases occur, it is still important for them to thus prepare themselves in order to *prevent* insanity, and to *arrest* it in its incipient stage.

We have no hesitation in saying, that if the pysicians of the country were fully aware of their duty to the community in this respect, and would exert themselves to prevent insanity by timely advice, and to arrest it in its early stage, that they would do those predisposed to insanity and the insane themselves, an amount of good unequalled by that of the asylums of the country.

They should understand and be able to recognize its earliest symptoms, for as has been said, insanity often, and we believe we may say most generally, exists in a slight and scarcely perceptible degree for months, before it is generally noticed. They should know how liable many are to this disease from hereditary predisposition, from previous attacks, long continued menorrhagia or other diseases, from repelled eruptions and extreme nervous susceptibility, and be able to advise such and warn them in time of impending danger. How many cases of puerperal insanity, or of that insanity that comes on after child-birth, might be prevented by timely precautions?

Physicians should study the various causes of mental diseases, and learn the danger of over-excitement of the nervous system, especially in early life by too strong emotions, by prematurely tasking the intellectual powers, by the improper indulgence of the appetites and passions, and by the neglect of moral and religious education; and thus be able to advise parents and others whenever they are pursuing a course likely to lead to this disease.

In this way, physicians would do great good in individual cases, and also very much towards arresting those alarming *epidemic delusions* that occasionally prevail through the country; a lamentable instance of which is within the knowledge of all, known by the name of "Millerism," or the doctrine of the immediate destruction of the world. This has not only sent many to the grave and rendered a vast number insane, but predisposed, we apprehend, a large number, by the excitement and terror it has produced, to nervous diseases and to insanity hereafter.

Physicians are often called upon to give their testimony in relation to the mental condition of individuals, and sometimes in cases where not only property but *life* is at stake. In such cases, their responsibility is very great, and furnishes strong additional reasons for their applying themselves diligently to the study of mental diseases.

The treatise of Esquirol on insanity has recently been translated and published in this country in a cheap form. It is a valuable book, and worthy of a place in the library of every physician. The works of Prichard and Combe, on the same subject, have also been republished here, and are considered standard authorities.—*Report of A. Brigham, Superintendant of the New York State Lunatic Asylum.*

